# Development and validation of osteoporosis prescreening model for Iranian postmenopausal women

**DOI:** 10.1186/s40200-015-0140-7

**Published:** 2015-03-17

**Authors:** Nassim Matin, Omidreza Tabatabaie, Abbasali Keshtkar, Kamran Yazdani, Mojgan Asadi

**Affiliations:** School of Medicine, Tehran University of Medical Sciences, Tehran, Iran; Osteoporosis Research Center, Endocrinology and Metabolism Clinical Sciences Institute, Tehran University of Medical Sciences, Tehran, Iran; Department of Epidemiology and Biostatistics, School of Public Health, Tehran University of Medical Sciences, Tehran, Iran; Endocrinology and Metabolism Research Center, Endocrinology and Metabolism Clinical Sciences Institute, Tehran University of Medical Sciences, Tehran, Iran

**Keywords:** Scoring, Logistic regression, Holdout validation, Prescreening, Osteoporosis

## Abstract

**Background:**

Studies have indicated that the commonly used osteoporosis prescreening tools are not appropriate for use in every nation. This study was designed to develop and validate a prescreening model for bone mineral densitometry among Iranian postmenopausal women.

**Methods:**

From 13613 individuals who were referred for bone mineral densitometry in Shariati hospital in Tehran, 8644 postmenopausal women were considered for the study after excluding men and premenopausal women. Questionnaires regarding the risk factors for osteoporosis were filled for each individual. Bone mineral density at the lumbar vertebrae (L2-L4), femoral neck and total femur was measured by dual X-ray absorptiometry. Using holdout validation, the study sample was divided into two parts; training set (5705) and test set (2939). Logistic regression analysis was performed on the training set. A scoring model was developed and tested in the test set.

**Results:**

Based on the training set, a seven-variable model named OPMIP (Osteoporosis Prescreening Model for Iranian Postmenopausal women) was developed with C statistics (area under curve) of 0.72. Using a cut-off of -2.5 for the model, the sensitivity, specificity, positive predictive value and negative predictive value were 72%, 59.5%, 64% and 69% respectively. The model performance was tested in the test set. OPMIP correctly classified 67.10% of cases with a sensitivity and specificity of 73.2% and 61%.

**Conclusions:**

In order to appropriately refer patients for a bone mineral densitometry, OPMIP can be used as a prescreening tool in Iranian Postmenopausal women.

## Introduction

Osteoporosis, the so-called silent disease, is the most common metabolic disease. Bones become fragile insidiously and as a result fractures with high morbidity and mortality rates, occur [1]. Because of the high morbidity and mortality affecting both the patient and the society, osteoporosis is often regarded as a global public health challenge. It is considered a health priority in Iran and a major contributor of the global burden of non-communicable diseases.

With growing numbers of the elderly in industrialized countries, the prevalence of osteoporosis and hip fractures increase. Already an established problem in the United States and European countries, it is appearing as a major public health problem in Asian countries as well. The growing number of the elderly and improved quality of life in these regions is a contributing main factor [2]. The incidence of hip fractures is estimated to rise from 1.66 million in 1990 to 2.26 million by 2050, of which 50% occur in Asia. Industrialized countries have a higher rate of osteoporotic hip fracture compared with that of developing countries. The rates are highest in Northern Europe and the US and lowest in Africa and Latin America. Although Iran is a developing country, the relative high rate of osteoporotic hip fractures is comparable to that of the US and other industrialized countries [3,4]. The age-standardized incidence of annual hip fractures in Iranian women is 7th in the world, higher than the US and many other European countries [5]. Iran contributes 0.85% and 12.4% of burden of hip fracture in the world and in the Middle East, respectively [6]. Geographical properties, genetics, ethnicities, latitude, population demographics and other environmental causes, influence osteoporosis prevalence and its complications.

Currently the best and most recommended method for diagnosing osteoporosis is bone mineral densitometry (BMD) using dual-energy X-ray absorptiometry (DXA). However DXA scan is not recommended as a screening tool for the whole population. Also according to the latest IOF (International Osteoporosis Foundation) audits, DXA is not available everywhere. Moreover, densitometry is overused in some areas and underused in some others, influenced by availability and socioeconomic factors [7].

With DXA being rather expensive and unavailable in some areas, developing prescreening tests, often referred to as the triage tests, is of benefit [8]. The first attempt to develop such a tool was performed by Slemenda in 1990 [9]. The model was later considered a poor model. Many other studies evaluated such models [10]. The most commonly used models in this regard are the SCORE (Simple Calculated Osteoporosis Risk Estimation) and the ORAI (Osteoporosis Risk Assessment Instrument) models, developed in 1998 by Lydick et al. and Cadarette et al. in 2000, respectively. They reported that SCORE is 89% sensitive and 50% specific, while ORAI is 95% sensitive and 41% specific [11,12]. Other models include Osteoporosis Self-Assessment Tool (OST), Study of Osteoporotic Fractures Simple Useful Risk Factors (SOFSURF), Osteoporosis Index of Risk (OSIRIS) and age, body size, no estrogen (ABONE) [13].

Based on previous studies, available prescreening models have not shown promising results in Iranian women [14]. Brief analysis in this data showed that these models are not sensitive enough. With regards to these analyses and unpublished academic theses, formerly proposed international models are not compatible with Iranian women; either these models should be calibrated or other prescreening tools should be developed [15].

The aim of the present study is to develop a simple and accurate prescreening tool for identifying patients at risk of osteoporosis who benefit from a DXA scan. So that osteoporosis screening is neither overused nor underutilized.

## Materials and methods

### Subjects

The study was conducted on 13613 who had undergone bone mineral densitometry in the BMD center of Shariati referral teaching hospital in the Iranian capital, Tehran, between April 2001 and April 2012. Patients referred for BMD, were referred by their family physicians or their specialists in the fields of endocrinology, rheumatology or nephrology.

Considering the low prevalence of Osteoporosis in men and premenopausal women, these groups were excluded from the study, leaving 8644 postmenopausal women, eligible for the study. The study was approved by the ethics committee of Endocrinology and Metabolism Research Institute affiliated to Tehran University of Medical Sciences.

### Measurements

A standard questionnaire on different osteoporosis risk factors was filled for every patient who was referred to this center. This questionnaire included details about demographic and anthropometric data (age, height, weight), gynecological and hormonal history (age at menarche, age at menopause, number of children, reproductive history), medical history (co-morbid conditions such as diabetes, rheumatoid arthritis, liver diseases, renal diseases, thyroid diseases, pathologic fractures, etc.) personal and family history of osteoporotic fractures, current lifestyle habits (physical activity and sunlight exposure, smoking, alcohol consumption and daily intake of dairy products) and concomitant medication use (glucocorticoids, diuretics, antipsychotics, antidepressive, antithrombotic and anticonvulsant drugs). Each participant completed a written informed consent and the ethics committee of Endocrine and Metabolism Research center in Tehran University of Medical Sciences approved this study.

Any physical activity performed for more than 30 minutes, at least three times a week, other than daily routines was defined as regular exercise. This included weight bearing exercises (such as jogging, walking and aerobic exercises) and resistance (such as weight lifting and body building) exercises. Appropriate walking was defined as walking at medium speed, at least 20 minutes, daily or at least more than three times a week. The use of medication or supplements was considered positive when used for more than three months. Dairy product intake was categorized into three groups: no servings, up to three servings, more than three servings. Use of vitamin D, Calcium supplements and hormone replacement therapy for more than three months were also considered.

Bone densitometer measurements were done using DXA machine (Lunar, 7164, GE, Madison, WI) in Shariati hospital BMD center. According to the World Health Organization (WHO); osteoporosis is defined as a BMD of at least 2.5 standard deviations below the mean for young adults of the same race and sex (T score). Osteopenia is defined as a T score of 1.0 to 2.5 SDs below the mean [16].According to most guidelines, the lumbar spine, femoral neck and total hip are regions of interest. (9) Most fractures occur with a T score that is 2 standard deviations (SDs) below the mean for young adults, thus the fracture threshold was defined and used [12,17-19].

A value of 2 or more SDs below the mean T score in either one of the lumbar spine, (L2 to L4 vertebrae) total hip or femoral neck, has been defined as a referral (fracture) threshold; the outcome of interest in this study.

### Statistical analysis

**Figure 1:**
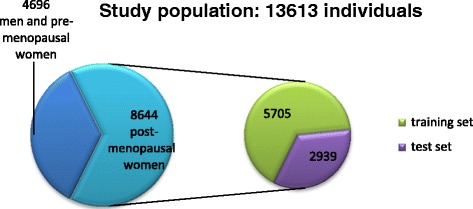
**The study population and how it got divided for analysis.**

After excluding men and premenopausal women, 8644 post-menopausal women were eligible for this study. “True validation” or “holdout validation” was used to evaluate the model. Using random sampling, the study sample was divided into two parts; 66%, equal to 5705 of the cases, called learning sample or training set, which were allocated to the development of the model as in true validation, and 33%; one third of the dataset, named test sample or test set (comprising 2939 individuals) allocated to validation of the model. This method is called the holdout method, the simplest cross validation [20]. A clear view is shown in Figure 1.

At first, descriptive analysis was performed on the whole dataset. After dividing the dataset into training and test set as mentioned above, analysis for developing the model in the training set begun. Chi-square was used to estimate the effect of each variable with the outcome (BMD of 2 or more SDs below the mean T score in either one of the three regions of interest) as the dependent variable. Logistic regression analysis with stepwise approaches was applied in the development of the model. We used β coefficient of each variable. Estimates were then rounded to the nearest integer and multiplied by 10, to develop a suitable scoring model. Using plotting receiver operating characteristic (ROC) curves, the area under the ROC curve, sensitivity, specificity, positive and negative predictive values and those correctly classified were determined at each threshold score. The area under curve, also called C statistic was estimated. A suitable cut-off point was selected with regards to sensitivity and specificity. The accuracy of the scores was then evaluated using the test set, which was left aside so far and not engaged in the model development process. In order to select and validate the final criteria for our scoring model, the model was applied to the test set, using ROC analyses. P-values less than 0.05 were considered statistically significant. The analysis was performed using STATA, version 11.2.

## Results

In order to categorize patients based on their osteoporosis results, T score was used in three regions; the lumbar vertebrae (L2-L4), the neck of femur and total hip. As already mentioned, osteoporosis is defined as a value of 2.5 standard deviations below the mean in any one of these three regions. Osteopenia or low bone mineral density is defined as the T score of 1 standard deviation or more below the mean. This classification is based on World Health Organization Diagnostic Criteria for Women without Fragility Fractures [16]. In this study, osteopenia is divided into two groups based on the value of 2 or more standard deviations below the mean which is defined as the fracture (referral threshold) [12,17-19]. Thus, two groups of osteopenic patients are defined: those who should be referred for DXA and those who shouldn’t. Others are classified as normal bone mineral density. Table 1 shows the frequency of the two osteopenic groups in both training and test set.

**Table 1:** **Frequencies of patients in each osteoporosis category based on T-scores in training and test sets**

**Training set**	**L2-L4**	**Femoral neck**	**Total femur**	**Either one of the regions**	**Important summations**
Normal T-score^a^	1548(28.37%)	1939(35.63%)	2479(45.54%)	1009^e^ (17.68%)	2643 (46.32%)^f^
Osteopenic but has not reached the fracture(refer) threshold^b^	1515(27.76%)	1867(34.31%)	1683(30.92%)	1634(28.64%)	
Osteopenic patients who should have been referred^c^	744(13.63%)	718(13.19%)	587(10.78%)	870(15.24%)	2825 (49.51%)^g^
Osteoporotic patients who should have been referred^d^	1650(30.24%)	918(16.87%)	694(12.75%)	1955(34.26%)	
**Test set**	L2-L4	Femoral Neck	Total Femur	Either one of the regions	Important summations
Normal T-score^a^	821(29.29%)	998(35.68%)	1307(46.75%)	531(18.06%)	1364 (46.41%)^f^
Osteopenic but has not reached the fracture(refer)threshol^d^	740(26.40%)	982(35.11%)	892(31.90%)	833(28.34%)	
Osteopenic patients who should have been referred^c^	417(14.88%)	395(14.12%)	291(10.41%)	483(16.43%)	1445 (49.16%)^g^
Osteoporotic patients who should have been referred^d^	825(29.43%)	422(15.09%)	306(10.94%)	962(32.73%)	

Notes:

^a^Normal = BMD value within 1 SD of the young adult mean.

^b^Those with a low BMD however have not reached the referral threshold. Meaning those with a BMD value of between −1 SD and −2 SD (Fracture or referral threshold) below the young adult mean.

^c^Those with a low BMD who should be referred. Meaning those with a BMD value of between −2 SD and −2.5 SD below the young adult mean.

^d^Those with BMD value of at least −2.5 below the young adult mean.

^e^A patient is considered as normal BMD when neither one of the regions has a BMD value of at least less than −1 SD below the young adult mean.

^f^This summation indicates patients below the referral threshold. (Negative outcome of interest).

^g^This summation indicates patients who should have been referred. (Positive outcome of interest).

BMD: Bone mineral Density, SD: Standard Deviation.

In this study, the dependent or outcome variable was BMD value of at least 2 or more standard deviations below the young adult T score mean. During the analysis, attempts were made for selection of the best form (categorized, dichotomous or linear) of each predictor variable. Those variables which were selected for the multivariate model, based on bivariate analysis, are shown in Table 2. The definition of variables is mentioned in the previous section. In this table, body mass index (BMI), age and years since menopause are categorized, however due to better performance of the model when linear; these variables were not categorized in the model. Ages of the patients ranged from 23 to 94 (mean ± standard deviation: 58.99 ± 8.69), menopause age ranged from 18 to 69 (47.07 ± 6.42) and BMIs ranged from 14.02 to 55.24 (27.91 ± 0.06).

**Table 2:** **Predictors of T score of ≤ 2 SDs in the training set**

**Training set**		**Number of patients who should be referred (%)**	**Number of patients with osteoporosis (%)**
Diabetes mellitus	Yes	307(10.86)	214(10.94)
	No	2518(89.13)	1741(89.05)
Appropriate walking	Yes	1196(42.33)	778(39.79)
	No	1629(57.66)	1177(60.20)
Regular exercise	Yes	126(4.46)	76(3.88)
	No	2699(95.53)	1879(96.11)
Using corticosteroids	Yes	360(12.74)	270(13.81)
	No	2465(87.25)	1685(86.18)
Body mass index	≤18.5	72(2.62)	65(3.41)
	18.5-25	880(32.10)	673(35.40)
	25-30	1171(42.72)	785(41.29)
	≥30	618(22.54)	378(19.88)
Years since menopause in 10 year group	10	944(33.59)	556(28.57)
	20	1094(38.93)	772(39.67)
	30	564(20.07)	439(22.55)
	40	183(6.51)	156(8.01)
	50	25(0.88)	23(1.18)
Age in 10 year group	20	2(0.07)	1(0.05)
	30	9(0.32)	5(0.25)
	40	200(7.11)	110(5.65)
	50	1024(36.44)	644(33.09)
	60	1020(36.29)	723(37.15)
	70	495(17.61)	411(21.12)
	80	60(0.21)	52(2.67)

Since in this study a large number of patients, with large number of variables were investigated, many predictor variables became statistically significant. So variables initially included in the multivariate analysis were chosen based on the results of bivariate analyses and clinical grounds. Complex variables were also excluded because this model is designed for a broad use and should be readily applied in different areas of the country. Seven predictor variables were considered for inclusion in this model. These predictors include age, body mass index, years since menopause, corticosteroid use (more than three months), diabetes, appropriate walking and regular exercise. Since these two variables are easily biased, we examined the model with and without these two variables, using logistic regression and backward approach. Table 3 presents the discriminatory performance of the models including 5, 6 and 7 variables. Finally, a seven-variable model was selected. Using categorized variables lowered the performance of the model.

**Table 3:** **Performance of different multivariate models based on the number of variables**

**Model**	**Area under curve**	**Sensitivity %**	**Specificity %**	**Positive predictive value %**	**Negative predictive value %**	**Correctly classified %**
1	0.66	56.00	69.25	64.31	61.44	62.69
2	0.68	57.76	70.69	66.08	62.86	64.26
3	0.71	62.39	69.52	66.69	65.18	65.88
4	0.71	62.02	70.19	67.19	65.24	66.14
5	0.72	63.01	69.76	67.22	65.70	66.41
6	0.72	63.04	70.11	67.50	65.83	66.61
7	0.72	63.07	70.04	67.54	66.03	66.75
8	0.72	63.23	70.08	67.57	65.93	66.68

1: age, 2: age and years since menopause, 3: age, years since menopause, BMI, 4:age, years since menopause, BMI and diabetes, 5:age, years since menopause, BMI, diabetes and corticosteroid use, 6:age, years since menopause, BMI, diabetes, corticosteroid use and appropriate walking, 7: age, years since menopause, BMI, diabetes, corticosteroid use and regular exercising. 8: age, years passed since menopause, BMI, diabetes, corticosteroid use, regular exercising and appropriate walking.

Table 4 presents the final model. Rounding the β coefficients for simplicity and multiplying them by 10; Our Osteoporosis Prescreening Model for Iranian Postmenopausal women (OPMIP) was developed.

**Table 4:** **The final results of the multivariate analysis and the developed scoring system (OPMIP)**

**Variable**	**β coefficient (95% CI)**	**Odds ratio (95% CI)**	**Standard error**	**P value**	**Score**
Age at entry	0.04 (0.02_0.05)	1.04 (1.03_1.05)	0.0052	0.000	0.4
Years passed since menopause	0.04 (0.03_0.05)	1.04 (1.03_1.05)	0.0051	0.000	0.4
Body mass index	−0.11 (−0.13_-0.10)	0.88 (0.87_0.90)	0.0068	0.000	−1
Diabetes mellitus	−0.42 (−0.60_-0.24)	0.65 (0.54_0.77)	0.0903	0.000	−4
Corticosteroid	0.60 (0.41_0.80)	1.83 (1.51_2.22)	0.0979	0.000	6
Regular exercise	−0.31 (−0.57_-0.05)	0.73 (0.56_0.94)	0.1325	0.018	−3
Appropriate walking	−0.20 (−0.32_-0.08)	0.81 (0.72_0.91)	0.0608	0.001	−2
Constant	0.45 (−0.19_1.1)				

**Figure 2:**
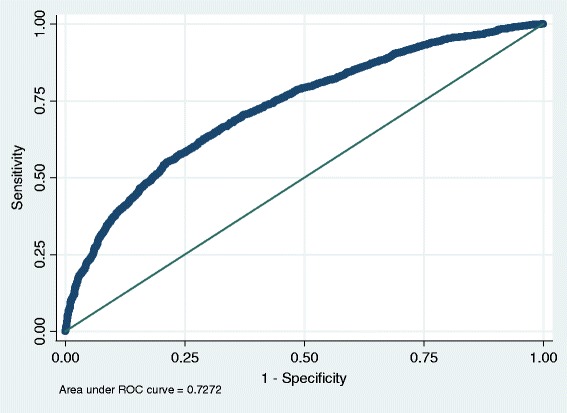
**The ROC curve for the OPMIP in the training set.**

ROC (receiver operating characteristic) curve analysis was performed on the final scoring model. The ROC curve for the model in the training set is shown in Figure 2. The full model has a C statistic (area under curve) of 0.72 (95% CI: 0.71-0.74) and a Hosmer Lemeshow statistic of 10.40 (P-value: 0.23). Based on the ROC curve analysis, a cut-off point of −2.5 was selected as appropriate for this model. The score of −2.5 correctly classifies 66% of patients with a sensitivity of 72.60% and specificity of 59.45%. The OPMIP score of −2.5 or more, identifies 72% of women with a BMD score of 2 or more SDs below the mean who should be referred for a DXA scan.

To this point, analyses were performed on the training set. Next, the model was internally validated using the test set. The performance of the model did not differ significantly in the training and test datasets. In the test sample, the model had a sensitivity of 73.2% and specificity of 61% and correctly classified 67.10% of cases. The performance of the model in the training and test datasets, considering the cut-off point, is shown in details in Table 5.

**Table 5:** **Performance of the OPMIP in the training and test samples**

**Samples**	**AUC**	**Sensitivity (95% CI) %**	**Specificity (95% CI) %**	**Positive predictive value %**	**Negative predictive value %**	**Correctly classifies %**
Training set	0.7273, (0.71-0.74)	72.6, (70.9-74.3)	59.5, (57.6-61.2)	64, (62.1-65.5)	68.8, (67–70.6)	66.68
Test set	0.7280, (0.70-0.74)	73.2, (70.8-75.5)	61, (58.4-63.6)	65, (62.6-67.4)	69.7, (67.1-72.2)	67.10

## Discussion

In this study, a prescreening seven-variable model was developed and tested. This model was 72% sensitive and accurately classified about 67% of patients.

Since the introduction of osteoporosis prescreening methods, several studies have been organized for triaging postmenopausal women for bone mineral densitometry, because screening all women is not cost-effective. All of these clinical stratification methods are intended to introduce a balance between the costs and the fact that anyone who might benefit from diagnosis should not be missed. Best validated tests are Osteoporosis Self-Assessment Tool, based on an Asian study population [21], the Osteoporosis Risk Assessment Instrument from a population-based Canadian cohort [12] and Simple Calculated Osteoporosis Risk Estimation from United States population [22]. However because of the varying performance of the original cut points proposed in different populations and lack of validation in different study groups, they have yet to be used internationally.

These models might not be applicable in every nation because of the multifactorial nature of osteoporosis. Factors such as genetics, ethnicity, geographic and cultural backgrounds, nutrition and different life styles are contributed to different images of osteoporosis in different areas. Thus the validity and accuracy of these models vary from country to country. The US preventive Services Task Force evaluated and reviewed these models, considering their methodological limitations such as lack of generalizability and lack of validation and concluded that these models should be further reviewed and approved [10]. A systematic review, evaluating 48 of such tools, of which 20 were externally validated, reported that only six tools were acceptable with regards to their method: OST, ORAI, Garvan, and SCORE, FRAX (WHO Fracture Risk Assessment Tool and Qfracture. This study concluded that none of these prescreening tools improved the selection of patients who would benefit from treatment [23].

Numerous studies have evaluated the use of these models in different ethnicities. One study revealed that the ORAI sensitivity ranged from 0.60 to 0.68 when used for nonHispanic white, African-American and Hispanic populations. The same study, reported a sensitivity of 0.80, 0.30 and 0.71 in the same population for SCORE [24]. In the Danish Osteoporosis Prevention Study, the sensitivity of ORAI, SCORE and OSTA ranged from 18 to 92% and the specificity ranged from 66 to 85%. They also reported a variation in different study sites; for instance, OST was 92% sensitive in the femoral region compared with 51% in the lumbar spine region [25]. Another population based study, using the area under curve (AUC) for discriminating between the prescreening tools, concluded an AUC of 0.70 for ORAI, SCORE, OSIRIS and OST, different from previous studies [26]. These studies support the idea that one model might not be applicable to all ethnicities and populations.

When SCORE was first developed and validated in 1998- based on femoral neck BMD of 2 or more SDs below the mean-scores higher than 6 had a sensitivity of 89%, a specificity of 50%, ROC area of 0.81 [11]. ORAI, developed in 2000, had a sensitivity of 95%, specificity of 41% when equal to or greater than 9 [12]. A sensitivity of 98% and a specificity of 29% were also reported for OST [21]. Other models with area under curve (AUC) s of ≤0.6 are also available. They are not classified in the “good” quality rated models [10,27].

Further studies were conducted to evaluate the performance of these models. Results revealed that these models have lower discriminating power than previously assumed. One study, designed to asses diagnostic accuracy of OST,ORAI and SCORE in women aged 67 years and more, concluded an AUC of 0.76, 0.70 and 0.73, sensitivities of ≥85% and specificities of ≤48%, for OST, ORAI and SCORE, respectively. In that study, the outcome variable was defined as the lumbar or femoral neck T score of 2.5 or more SDs below the mean T score [28]. A Korean study, yielded AUCs of 0.79,0.79,0.76 and 0.78 for OST,ORAI, SCORE and OSRIRIS, respectively. Sensitivities ranged from 0.65 to 0.76 [29]. One study revealed that OST performs poorly for predicting osteoporosis in the lumbar spine and moderately in the femoral neck, which yields to different sensitivities and specificities based on the outcome of interest [30]. Another study, showed a sensitivity of 68% for ORAI and 54% for SCORE in overall accuracy evaluating Hispanic, NonHispanic and African-American women. The AUC was 74% for ORAI and 67% for SCORE [24].

One study in Iran showed acceptable performance of OST and ORAI, with sensitivity and specificity of around 70% and 60% respectively [31]. Another study in Iran indicated that a large number of bone mineral densitometries (BMDs) performed based on the preformed prescreening models, were not necessary. It indicated that OST and ORAI lead to 60% and 50% unnecessary DXA scans, respectively. It also reported that only 50% of the BMDs were appropriate based on SCORE and ORAI. International Osteoporosis Foundation guidelines conducted only 33.3% appropriately. About 40% of BMDs were appropriately performed using OST and OSIRIS models and only 10%, based on ABONE. So, these prescreening tools are not suitable for Iranian population and relying on them leads to unnecessary tests around the country [14]. Applying these models to the data used in this paper was not an objective of this study; however a rough estimate in the early analyses phase, not published yet, revealed that applying OST, SOFSURF and OSIRIS, has a sensitivity of only 30 to 50%. ABONE, SCORE and ORAI were more compatible with this data, being around 60 to 70% sensitive, with low specificity and low Positive and Negative Predictive Values, which are important characteristics of a risk assessment tool. The AUC for these models, ranged from 0.57 to 0.68, confirming that they are not suitable decision making tools for a DXA scan. This analysis showed that these models lack the sensitivity for a “good” prescreening tool. Not being representative of every population in the world, national attempts should be made to develop a suitable model for each nation. Studies have also concluded that using these models have caused unnecessary DXA scans, not only in Iran but also in many other countries [7,14,32].

Many reasons explain this varied performance. First the dependent (outcome) variables used for stratifying patients are different in these prescreening models. That is; while the OST uses femoral neck T score equal to or less than −2.5, ORAI uses femoral neck or lumbar spine T score equal to or less than −2 and SCORE uses femoral neck T score equal to or less than −2 as dependent variable. This inconsistency leads to variable performance. Recently, osteoporosis is defined based on bone mineral density in either one of these three regions of interest: Femoral neck, lumbar spine or the total femur area [33]. Moreover, these models should be used for prophylactic maneuvers and treatment threshold is far from prescreening goals. It means that T-score of ≤ −2.5 is too late for prophylactic maneuvers. This is while the Fracture Threshold (T score of 2 or higher) is an acceptable alternative in this regard and can be used for screening women for DXA. Another limit for these models is the use of hormone replacement therapy (HRT). In fact, when ORAI and SCORE were proposed, HRT use was prevalent because of highly positive attitude towards them, which proved to be overly optimistic later [34,35]. HRT is not prevalent these days; a finding also confirmed in this study. Other limits for these models, is the overlooked effects of environment and life style on bone health. Life styles, habits and diets vary significantly among the different areas of the world. Some studies have reported different anatomical sites of osteoporosis across different racial groups, for example the lumbar spine is more likely to be involved in osteoporosis than the hip in African-American women [36].

In this study, for the first time, we developed a model based on logistic regression analysis and holdout method using a group of postmenopausal women. The holdout method was used, dividing the study sample (8644 postmenopausal women), into two samples, one to develop the model (5705 women) and the other one for testing the model (2939). (Internal Validity) The cut off of −2.5 was calculated for the model named OPMIP. In other words, individuals with OPMIP score of more than −2.5 should be referred for DXA. The area under curve was 0.72 which is considered “good” for a prescreening or screening model. The Hosmer Lemeshow statistic of 10.40 indicated that the model is well calibrated. Our model has a sensitivity of 72% and specificity of 60%, correctly classifying 66% of cases. The positive and negative values of our model were 64% and 68% respectively.

Although this study has many positive points such as the large sample size, limits are yet to be confronted. The studied population might not be representative for the whole Iranian population, since BMD center was located in a referral hospital in Tehran, the capital of the country. In fact, participants who are referred for BMD measurement might have more risk factors (like the use of glucocorticoids) than postmenopausal women in the general population. Also appropriate walking and regular exercise, which are proposed as independent variables in this study, were assessed based on self-reports. The fact that the outcome is based on the fracture threshold in three regions is a double-sided sword. The positive point is the accuracy and the correct definition of outcome. However, this model is not totally comparable to others because of different dependent variables. Further studies should be done to evaluate and compare this model with other available models. Also, the holdout method might not be efficient for estimating internal validity and might be weaker than other cross validation methods [37]. Another limit is that we have not examined our model in other study population. We can’t conclude that this method is good enough for all rural and urban areas and family medicine centers. The model might be too complex for a nationwide use. Attempts should be made to develop a simple algorithm for this method to make it feasible in every medical center. The development of other statistical softwares and better statistical methods might also provide a better decision tool for this purpose. One point is important however, that financial limits highlight the need of simple prescreening models for DXA testing which is the ultimate purpose in this study and many similar studies.

## Abbreviations

BMD: Bone mineral densitometry
DXA: Dual-energy X-ray absorptiometry
IOF: International Osteoporosis Foundation
SCORE: Simple Calculated Osteoporosis Risk Estimation
ORAI: Osteoporosis Risk Assessment Instrument
OST: Osteoporosis Self-Assessment Tool
SOFSURF: Study of Osteoporotic Fractures Simple Useful Risk Factors
OSIRIS: Osteoporosis Index of Risk
ABONE: Age, body size, no estrogen
SD: Standard deviation
ROC: Receiver operating characteristic
BMI: Body mass index
OPMIP: Osteoporosis Prescreening Model for Iranian Postmenopausal women
FRAX: WHO Fracture Risk Assessment Tool and Qfracture
WHO: World health organization
AUC: Area under curve
HRT: Hormone replacement therapy
